# Genetic and Chemical Evaluation of *Trypanosoma brucei* Oleate Desaturase as a Candidate Drug Target

**DOI:** 10.1371/journal.pone.0014239

**Published:** 2010-12-06

**Authors:** Andrés Alloatti, Shreedhara Gupta, Melisa Gualdrón-López, Mariana Igoillo-Esteve, Paul A. Nguewa, Gladys Deumer, Pierre Wallemacq, Silvia G. Altabe, Paul A. M. Michels, Antonio D. Uttaro

**Affiliations:** 1 Facultad de Ciencias Bioquímicas y Farmacéuticas, Instituto de Biología Molecular y Celular de Rosario, CONICET, Universidad Nacional de Rosario, Rosario, Santa Fe, Argentina; 2 Research Unit for Tropical Diseases, Laboratory of Biochemistry and de Duve Institute, Université catholique de Louvain, Brussels, Belgium; 3 Department of Clinical Chemistry, Cliniques universitaires Saint-Luc, LTAP, Université catholique de Louvain, Brussels, Belgium; Universidade Federal de Minas Gerais, Brazil

## Abstract

**Background:**

Trypanosomes can synthesize polyunsaturated fatty acids. Previously, we have shown that they possess stearoyl-CoA desaturase (SCD) and oleate desaturase (OD) to convert stearate (C18) into oleate (C18:1) and linoleate (C18:2), respectively. Here we examine if OD is essential to these parasites.

**Methodology:**

Cultured procyclic (insect-stage) form (PCF) and bloodstream-form (BSF) *Trypanosoma brucei* cells were treated with 12- and 13-thiastearic acid (12-TS and 13-TS), inhibitors of OD, and the expression of the enzyme was knocked down by RNA interference. The phenotype of these cells was studied.

**Principal Findings:**

Growth of PCF *T. brucei* was totally inhibited by 100 µM of 12-TS and 13-TS, with EC_50_ values of 40±2 and 30±2 µM, respectively. The BSF was more sensitive, with EC_50_ values of 7±3 and 2±1 µM, respectively. This growth phenotype was due to the inhibitory effect of thiastearates on OD and, to a lesser extent, on SCD. The enzyme inhibition caused a drop in total unsaturated fatty-acid level of the cells, with a slight increase in oleate but a drastic decrease in linoleate level, most probably affecting membrane fluidity. After knocking down OD expression in PCF, the linoleate content was notably reduced, whereas that of oleate drastically increased, maintaining the total unsaturated fatty-acid level unchanged. Interestingly, the growth phenotype of the RNAi-induced cells was similar to that found for thiastearate-treated trypanosomes, with the former cells growing twofold slower than the latter ones, indicating that the linoleate content itself and not only fluidity could be essential for normal membrane functionality. A similar deleterious effect was found after RNAi in BSF, even with a mere 8% reduction of OD activity, indicating that its full activity is essential.

**Conclusions/Significance:**

As OD is essential for trypanosomes and is not present in mammalian cells, it is a promising target for chemotherapy of African trypanosomiasis.

## Introduction

Trypanosomes are parasitic flagellated protists belonging to the family Trypanosomatidae (order Kinetoplastida) of which many members cause human diseases. *Trypanosoma brucei* is responsible for human sleeping sickness in sub-Saharan Africa and *Trypanosoma cruzi* causes Chagas' disease in Latin America. Few drugs are available against these diseases and those currently used are toxic and the parasites are susceptible to generate resistance against them. It makes urgent the development of more safe and efficacious drugs, as well as the identification of new targets.

*T. brucei* multiplies extracellularly in mammals as the bloodstream form (BSF) of the parasite. In advanced stages of the disease, trypanosomes traverse the blood-brain barrier and invade the cerebrospinal fluid. The long-slender BSF differentiates into a non-replicative short-stumpy form that, upon ingestion by the tsetse fly during a blood meal, differentiates into the procyclic form (PCF) that dwells into the insect's midgut. Inside the insect, the PCF changes successively into various other developmental stages while moving from the gut to the salivary glands. Finally, the parasite can be transmitted to a new mammalian host as the metacyclic form, with the saliva of the tsetse during another blood meal [1]. Cells of the metacyclic form and BSF are densely covered with a surface coat made up of a single type of variant surface glycoprotein (VSG). The regular switching to the expression of a different VSG (“antigenic variation”) allows the parasite population to evade the immune system [2].

For many years it was known that *T. cruzi* and PCF *T. brucei* are capable of synthesizing fatty acids (FA), but it was believed that BSF African trypanosomes were entirely dependent on their host for their lipid content. However, recently it was reported that in addition to the uptake of host lipoproteins, BSFs are also able to synthesize *de novo* their own FAs through an unprecedented mechanism [3]. Short- and medium-chain FAs are usually synthesized by means of the soluble fatty-acid synthetase (FAS) systems and subsequently elongated to long and very long FAs by the particulate elongase (ELO) system that resides in the endoplasmic reticulum [4]. Trypanosomatids lack the classic eukaryote or type I FAS but conserve a mitochondrial type II FAS [5]. This latter system is mainly involved in the production of octanoate for lipoic acid synthesis and contributes only to a small fraction of total FA biosynthesis for use in the mitochondrion. The main FA pool is synthesized by a specialized ELO system that appears to be an adaptation to the parasitic lifestyle [6]. *T. brucei* has three ELOs responsible for the successive elongation of butyryl-CoA to decanoyl-CoA (ELO1), then to myristoyl-CoA (ELO2) and finally to stearoyl-CoA (ELO3). This modular synthesis allows the parasite to regulate the production of different intermediates as required throughout its life cycle. For example, by repression of ELO3 the BSF accumulates myristoyl-CoA, to supply part of the paramount requirements of C14 moieties in the synthesis of the glycosylphosphatidylinositol (GPI) anchor of VSGs [6], [7]. *T. cruzi* has an additional ELO (ELO4) [6], [8] which elongates C18, and probably C16, to C24 and C26 FAs (Livore and Uttaro, unpublished results), required in the synthesis of anchors for surface macromolecules, such as mucins and glycoinositol phospholipids. The ELO pathway and the mitochondrial FAS appear to be essential in *T. brucei*, consequently being potential targets for the development of new trypanocidal drugs. For instance, the antibiotic thiolactomycin seems to inhibit both pathways and kills cultured parasites with an EC_50_ of 150 µM [6].

The stearate (C18:0) synthesized by the trypanosomatid ELO system is mainly converted into linoleate (C18:2). Linoleate represents near 30% and 40% of total FAs in *T. brucei* and *T. cruzi*, respectively, being the principal acyl moiety in membrane phospholipids. Total unsaturated FAs can represent up to 70% of FAs depending of the parasite species and life cycle stage [9], [10]. We have recently described the pathway of polyunsaturated FA (PUFA) biosynthesis in trypanosomatids. *Leishmania major*, also belonging to the Trypanosomatidae, has a complete set of eight enzymes that allow the parasite to synthesize C22:5 and C22:6 PUFAs from C18:0. By contrast, *T. brucei* and *T. cruzi* desaturate C18:0 to oleate (C18:1) by means of the stearoyl-CoA Δ9 desaturase (SCD) and then to C18:2 by the oleate Δ12 desaturase (OD) [11]. Linoleate cannot be further desaturated nor elongated as trypanosomes lack the enzymes linoleate Δ15 desaturase, Δ6 C18 desaturase, Δ6 PUFA ELO and Δ5 C20 desaturase. However, up to 20% of the trypanosome's total FAs are C22 PUFAs. These are synthesized from the intermediates arachidonate (C20:4n6) and eicosapentaenoate (C20:5n3) taken up from the host, which are elongated by a Δ5 PUFA ELO and desaturated by a Δ4 C22 desaturase [8], [12]. Such proportion of unsaturated FAs could provide the cell with a high membrane fluidity that may be essential for the parasites to adapt themselves to the dramatic changes in temperature and chemical parameters experienced during their complex life cycles. For this reason we speculated that the biosynthesis of linoleate has to be essential in trypanosomes. In addition, OD is not present in mammals highlighting this enzyme as a putative selective chemotherapeutic target. We have recently shown that oleate and linoleate biosynthesis can be specifically inhibited in *T. cruzi* [13]. Isoxyl (Thiocarlide) and 10-thiastearate (10-TS) inhibited the growth of the parasite with EC_50_ values of 2 and µM, respectively, due to the specific inhibition of SCD, reducing the level of oleate and, as a consequence, also linoleate in the membrane. Moreover, OD was specifically inhibited by 12- and 13-TS isomers, producing a drastic drop in both the linoleate content of *T. cruzi* epimastigotes and in parasite growth (with EC_50_ values of 50 and 10 µM, respectively). TS positional isomers are analogues of C18:0 with sulphur atoms substituting methylene groups in the carbon chain. As introducing sulphur atoms has little effect on the structure of the aliphatic chain, thia fatty acids are metabolized as ordinary FAs and incorporated into different lipid classes [14]. 9- and 10-TS were shown to be converted to the corresponding acyl-CoAs and to bind to hepatocyte's SCD, causing strong inhibition of Δ9 desaturation [15]. The same compounds had been earlier tested on cultures of the trypanosomatids *Crithidia fasciculata* and *Leishmania sp*., although as inhibitors of dihydrosterculic acid biosynthesis. This cyclopropane FA is exclusively synthesized by eukaryotic microbes such as species of *Crithidia*, *Herpetomonas*, *Leptomonas* and *Leishmania*, but not by trypanosomes or vertebrates, suggesting dihydrosterculic acid biosynthesis as a putative target for selective chemotherapy of leishmaniasis [16], [17]. However, our results (as obtained with *T. cruzi*) suggested that the deleterious effect, at least for 10-TS, was most probably due to a specific inhibition of the SCD and a consequent drop in levels of essential FAs such as oleate and linoleate, in the *Leishmania*'s membranes [13].

In the work presented in this paper we show that, like in *T. cruzi*, *T. brucei* OD can be inhibited by 12- and 13-TS in both PCF and the infective BSF, with a consequent drop in linoleate content and growth inhibition. Knock-down of OD gene expression by RNA interference (RNAi) in *T. brucei* PCF and BSF produced similar decreases of linoleate content and growth rate to that found upon administering TSs. It validates OD as a promising target for the development of selective chemical intervention.

## Materials and Methods

### Materials

Stearate, linoleate, oleate and sodium methoxide were obtained from Sigma-Aldrich (St. Louis, MI, USA). All organic solvents were purchased from Merck (Whitehouse Station, NJ, USA). Thiastearic acid positional isomers were synthesized as previously described [18], using reagents purchased from Sigma-Aldrich. For argentation-thin layer chromatography (argentation-TLC), silica gel plates (containing 10% silver nitrate) were purchased from Analthech (Analthech, Inc., Newark, DE).

### Trypanosomes, growth conditions and transfection

Bloodstream and procyclic forms of *T. brucei* Lister 427, cell lines 90-13 and 29-13, respectively [19] that were used in this study, harbour chromosomically integrated constructs with the genes of the T7 RNA polymerase and the *Escherichia coli* tetracycline (tet) repressor. The parasites were always cultured in the presence of G418 and hygromycin in order to maintain the respective constructs. BSF were cultured in HMI-9 medium containing 10% heat-inactivated foetal calf serum (Invitrogen) and 2.5 µg/ml G418 (Invitrogen) at 37°C under water-saturated air with 5% CO_2_. PCF were grown in SDM-79 medium [20] supplemented with 15% foetal calf serum and 50 µg/ml hygromycin (Sigma-Aldrich) and 15 µg/ml G418 at 28°C under water-saturated air with 5% CO_2_. Cultures were always harvested in the exponential growth phase, *i.e*., at densities lower than 2×10^6^ cells/ml for BSF and 2×10^7^ cells/ml for PCF, by centrifugation at 1,000×*g* for 10 min.

Transfection of trypanosomes and selection of clones were performed as described previously [21]. After transfection and selection, clones harbouring the recombinant construct used in the transfection were stored at −80°C in appropriate medium containing 12% glycerol.

Thiastearates, linoleate and oleate were added to the cultures as solutions in ethanol. The final concentration of ethanol in the cultures was always adjusted to 1%. Cultures were propagated by dilution with fresh medium with the corresponding concentrations of drugs. Cells were counted immediately before dilutions by using a Neubauer chamber. Growth curves were plotted as the product of cell density and total dilution versus time. Although no significant effect of ethanol was seen on growth, we performed for each experiment a control in which only the solvent was added to the culture. The growth curves were highly reproducible. Figures were drawn by using the mean ± SD values of three independent experiments. EC_50_ indicates the concentration of drug required to cause 50% inhibition of the growth rate. Although the indicated EC_50_ values were calculated by using the last points of the growth curves, almost identical values were found irrespective the day of treatment used to determine them.

### Fatty-acid analysis

Cells (2×10^8^) in the late logarithmic phase of growth were collected by centrifugation and the pellets washed twice with 8 ml of isotonic saline solution. Lipids were extracted according to Bligh and Dyer [22]. The organic phase was reduced to dryness under N_2_, and FA methyl esters were prepared by adding 1 ml of 0.5 M sodium methoxide in methanol and incubating for 20 min at room temperature. After neutralization with 6 M HCl and extraction with 2 ml hexane, the organic solvent was evaporated to dryness under a N_2_ stream. FA methyl ester composition was analyzed with a polyethylene glycol column (PE-WAX, 30 m×0.25 mm inside diameter, Perkin Elmer, Norwalk, CT, USA) in a Perkin Elmer AutoSystem XL gas chromatograph. Gas chromatographic analysis was performed at 180°C isothermically. The GC-MS was carried out using a Perkin Elmer mass detector (model TurboMass) operated at an ionization voltage of 70 eV with a scan range of 20–500 Da. The retention time and mass spectrum of any new peak obtained was compared with that of standards (Sigma-Aldrich) and those available in the database NBS75K (National Bureau of Standards). Percentages of FAs were calculated after integration of the chromatogram peaks. Figures were drawn by using the mean ± SD values of three independent experiments.

### Whole-cell radiolabeling and analysis of fatty acids

*T. brucei* cells in the logarithmic phase of growth were collected and resuspended in the same medium without serum at 10^7^ cells/ml. One ml of cell suspension was incubated at 20°C after addition of 5 µl of [1-^14^C]-stearic acid or [1-^14^C]-oleic acid (American Radiolabeled Chemicals Inc., St. Louis, MO) to a 70 µM final concentration (specific activity 36 mCi/mmol) in fatty-acid free albumin (1% final concentration), and 5 µl of desaturase inhibitor or the same volume of ethanol. The incubation was stopped by addition of 6 ml of chloroform/methanol (1∶2 v/v). After 2 min of vortexing, 2 ml of water and 2 ml of chloroform were added, vortexed again and centrifuged for 5 min at 2,000×*g*. The organic phase was recovered and washed with 4 ml of 2 M KCl by fraction partition, dried under a N_2_ stream and treated as described before, to obtain the FA methyl esters by transesterification. Methyl esters were subjected to argentation-TLC, using toluene as developing solvent [23]. Radioactivity was detected on a Typhoon 9200 PhosphorImager and, after the spots had been scraped from the plate, quantified by liquid scintillation radioassay.

### RNA interference

The *T. brucei* specific vector pZJM [24] was used to generate stable cell lines of both PCF and BSF trypanosomes for the tet-inducible expression of double-stranded RNA of OD. Double-stranded RNA was produced by transcription of a construct comprising a fragment representing the first half of the coding region of the gene (GenBank ID: AY372529). To minimize the possibility of nonspecific, off-target RNA degradation the gene fragment selected for making the RNAi construct was checked by performing a BLAST search against the *T. brucei* genome database. No DNA sequences with significant similarity were found. The construct was prepared by PCR amplification using the following oligonucleotides: forward primer 5′–ATAAGCTTATGTTGCCTAAGCAACAG–3′ (*Hind*III site underlined) and reverse primer 5′–CCCTCGAGGGTGTGTTTATGGTGAGT–3′ (*Xho*I site underlined). The sequence of the amplified DNA was checked before it was inserted between the two tet-inducible T7 promoters of vector pZJM, containing a gene for phleomycin resistance (*ble*). The linearized recombinant pZJM-OD plasmid was targeted for integration by homologous recombination into the transcriptionally silent ribosomal RNA gene repeat spacer of the *T. brucei* genome. Transfections of trypanosomes and selection of clones were performed as described, using 1.25 and 2.5 µg/ml phleomycin (Sigma-Aldrich) [24], respectively for BSF and PCF [21], [24], concentrations at which all wild-type cells died within 12 days, whereas a few clones could be obtained that survived and grew normally during long-term cultivation. In order to confirm the genomic integration of the plasmid, DNA was isolated from the selected BSF OD RNAi cell line (propagated for many generations after transfection with the not-autonomously replicating plasmid) as well as the non-transfected (wild-type) BSF 90.13 and PCF 29.13 lines, using the PureLink Genomic DNA mini kit (Invitrogen). Genomic integration of the pZJM-*OD* DNA was then checked by PCR, by amplifying two segments of the recombinant plasmid, a 252 bp segment of the phleomycin resistance conferring *ble* gene (GenBank ID: X52869) and an approximately 1 kb DNA fragment containing parts of both the *OD* and *ble* genes and the intergenic region. To that end, the *ble*-specific forward primer 5′–AGTTGACCAGTGCCGTTCC–3′ and reverse primer 5′–CGGAAGTTCGTGGACACGA–3′ were used and the *OD* gene specific forward primer 5′–GACATGTTTCAGCTTTTCCTC–3′ and the *ble*-specific reverse primer 5′–CGGAAGTTCGTGGACACGA–3′. The PCR reactions were performed using the *GoTaq* DNA polymerase (Promega) at an annealing temperature of 60°C and with 35 cycles. Amplified products were analyzed by electrophoresis on 1% agarose gels. No amplification products were obtained when wild-type cell DNA was used in the PCR reactions.

For induction of double-stranded RNA, PCF and BSF cells were cultured in appropriate medium containing 1 µg/ml of tet. To assess the RNA knockdown, a semi-quantitative assay for mRNA was performed. Parasites were harvested from 20 ml of a PCF culture (10^7^ cells/ml) and 100 ml of a BSF culture (10^6^ cells/ml) after induction with tet at each 24 h during four days. Cells were collected by centrifugation at 2,000×*g* and washed twice with 10 ml of ice-cold phosphate buffered saline. Wild-type PCF and BSF cells were used as controls. Total RNA was extracted using the SV Total Isolation System Kit of Promega and subsequently cDNAs were synthesized with the RevertAid H Minus First Strand cDNA Synthesis Kit of Fermentas. These cDNAs were synthesized using appropriate primers: for *OD* the forward and reverse primers were 5′–TAATGCCATTGAGGTAAC–3′ and 5′–TGCGAGTAATGCAGATCC–3′, respectively. Tubulin was amplified for normalization purpose utilizing the oligonucleotides 5′–GCGCGAAATCGTCTGCGTTCAGG–3′ and 5′–GCACGTACGGAGTCCATTGTACC–3′ as forward and reverse primers, respectively. PCR experiments were performed with the *GoTaq* DNA polymerase (Promega) at 58°C using 18 cycles for BSF, 26 cycles for PCF and 18 cycles for the tubulin amplification (the number of cycles were chosen in order to avoid saturation of the signal). The amplified fragments were resolved on 2% agarose gels and subsequently visualized by treatment for 20 min with Sybr Safe DNA Gel Staining (Invitrogen). The gel was then scanned with a Kodak image station 2000MM and the relative amount of amplification products was analyzed by densitometry. This procedure was calibrated to assure a signal proportional to the amount of DNA. The intensity of the bands of interest was corrected by the intensity of the tubulin band amplified from the same cDNA. Results obtained for RNAi induced cells were expressed as percentages of those obtained for wild-type cells.

## Results

### Effect of 12- and 13-thiastearates on procyclic-form *T. brucei*

**Figure 1:**
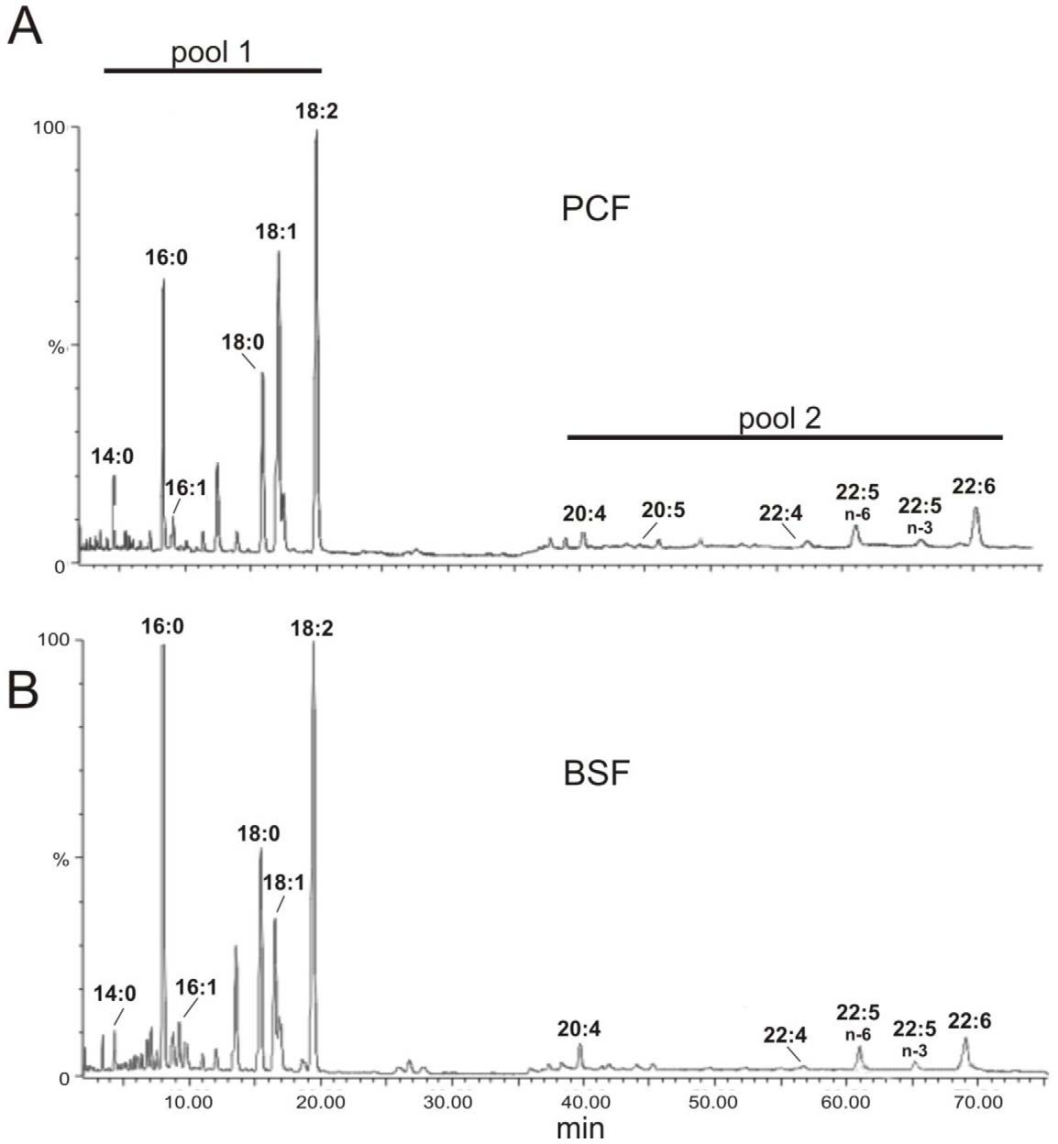
Gas chromatography profile of total fatty acids from *Trypanosoma brucei* cells. Procyclic form (PCF) (A). Bloodstream form (BSF) (B). Pool 1 indicates those fatty acids which are *de novo* synthesized by the parasite cells and partially taken up from the culture media; pool 2 indicates fatty acids taken up from the culture media plus those resulting from their elongation and desaturation.

**Figure 2:**
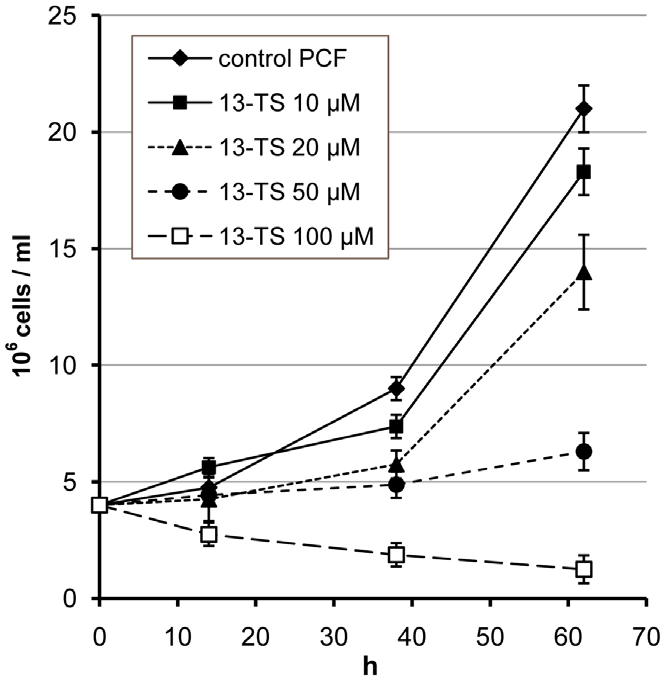
Effect of 13-thiastearate on the growth of procyclic-form *Trypanosoma brucei*. Growth curves are presented of cells cultured in the absence (control) or presence of increasing concentrations of 13-thiastearate (13-TS). The results are means ± SD of three independent experiments.

We have previously identified and characterized the OD of *T. brucei* [11]. The enzyme is a typical methyl-end desaturase, highly similar to fungal and plant ODs [25]. Its gene (GenBank ID: AY372529, protein ID: AAQ74969; GeneDB ID: Tb927.2.3080, http://www.genedb.org) was expressed in *Saccharomyces cerevisiae*, where the enzyme showed an absolute requirement for cytochrome *b_5_* as electron donor and a high specificity for oleate as substrate. An important role of OD in *T. brucei* is in agreement to the FA profiles shown in Fig. 1 and Table 1, with C18:2 representing 30% of total FAs in PCF and 34% in cultured BSF; other di-unsaturated FAs represent less than 1% of total FAs. The *T. cruzi* (AAR23833) and *L. major* (CAJ06920) orthologues share 61% and 58% of identity, respectively, with *T. brucei* OD. We have shown recently that OD activity in *T. cruzi* can be specifically inhibited by 12- and 13-TS, which is most probably the reason of the growth inhibition of epimastigote (i.e. insect stage) cultures, due to a drastic reduction of the essential FA linoleate in the parasite membrane [13]. The high similarity between ODs of both trypanosome species prompted us to assay the toxicity of TS isomers on *T. brucei* PCF cultures. As shown in Fig. 2, growth of PCF was totally inhibited by 100 µM 13-TS, with an EC_50_ of 30±2 µM. 12-TS has the same effect (EC_50_ of 40±2 µM) which was not reverted by supplementing the medium with oleate or linoleate up to 1 µM; higher concentrations of FAs cannot be assayed as they are toxic even in the control experiment without inhibitor (data not shown). Such toxic effect of free FAs was not observed on *T. cruzi* epimastigotes [13]. After 30 h of growth in the presence of 100 µM TSs, the FA profiles of the cells were determined by GC-MS. A notable drop in linoleate level was found, from 30.7% (control) to 13.3% (12-TS) and 11.9% (13-TS) (Table 1). The levels of oleate and palmitoleate (C16:1) did not show major variations whereas stearate slightly increased from 10.4% to approximately 13%. The conversion of oleate to linoleate was reduced from 65% to 38–42% which is indicative of *T. brucei* OD inhibition. However, an inhibitory effect on SCD cannot be ruled out, as the cells maintained normal levels of oleate, which was expected to be increased after OD inhibition. Palmitate (C16:0) was importantly increased, which could be an adaptation of the cell in order to maintain a normal membrane fluidity, by replacing unsaturated FAs, which dropped from 49% to 33–34%, by shorter saturated FAs; a similar effect was previously also found in *T. cruzi* [13]. A 40% reduction in linoleate content was only seen after treatment with 20 µM of 13-TS for 14 h (data not shown).

**Table 1:** Relative fatty-acid composition of procyclic and bloodstream forms of *Trypanosoma brucei*.

Fatty acid	PCF			BSF
	control	12-TS	13-TS	
14:0	0.3±0.1	2.3±0.2	1.7±0.1	0.9±0.1
16:0	8.2±3.1	20.9±2.3	20.4±2.5	14.9±2.1
16:1	1.7±0.1	2.6±0.4	2.3±0.8	1.7±0.4
17:1	3.0±1.1	2.9±0.8	2.2±0.4	1.4±0.1
18:0	10.4±0.5	12.7±1.1	13.0±2.4	14.8±1.2
18:1 Δ9	16.5±2.1	18.2±1.9	18.8±1.3	11.1±0.9
18:1 Δ11	4.2±0.2	4.1±0.3	3.6±0.2	3.8±0.4
18:2	30.7±1.6	13.3±1.5	11.9±0.4	34.0±3.1
20:0	<0.2	-	-	0.6±0.2
20:1	0.3±0.1	0.4±0.2	0.4±0.1	0.8±0.1
20:2	0.7±0.4	2.6±0.8	2.2±0.6	0.4±0.2
20:3 n-6	1.6±0.7	1.9±0.4	2.1±0.3	0.6±0.1
20:3 n-3	0.20±0.01	-	-	0.6±0.2
20:4 n-6	2.0±0.5	3.0±0.7	2.8±0.3	1.9±0.4
20:5 n-3	<0.2	-	-	-
22:4 n-3	1.7±0.4	0.2±0.1	1.3±0.1	0.7±0.2
22:5 n-6	5.4±2.1	3.0±0.6	3.7±0.5	3.8±1.1
22:5 n-3	1.2±0.3	1.7±0.2	1.5±0.3	1.1±0.3
22:6	11.7±4.5	9.4±2.7	12.0±1.8	6.3±1.8

Fatty acid composition (%) of procyclic form (PCF) was analysed before and after treatment with 100 µM thiastearate isomers (12-TS and 13-TS). BSF, bloodstream form.

### RNA interference of oleate desaturase in procyclic-form *T. brucei*

**Figure 3:**
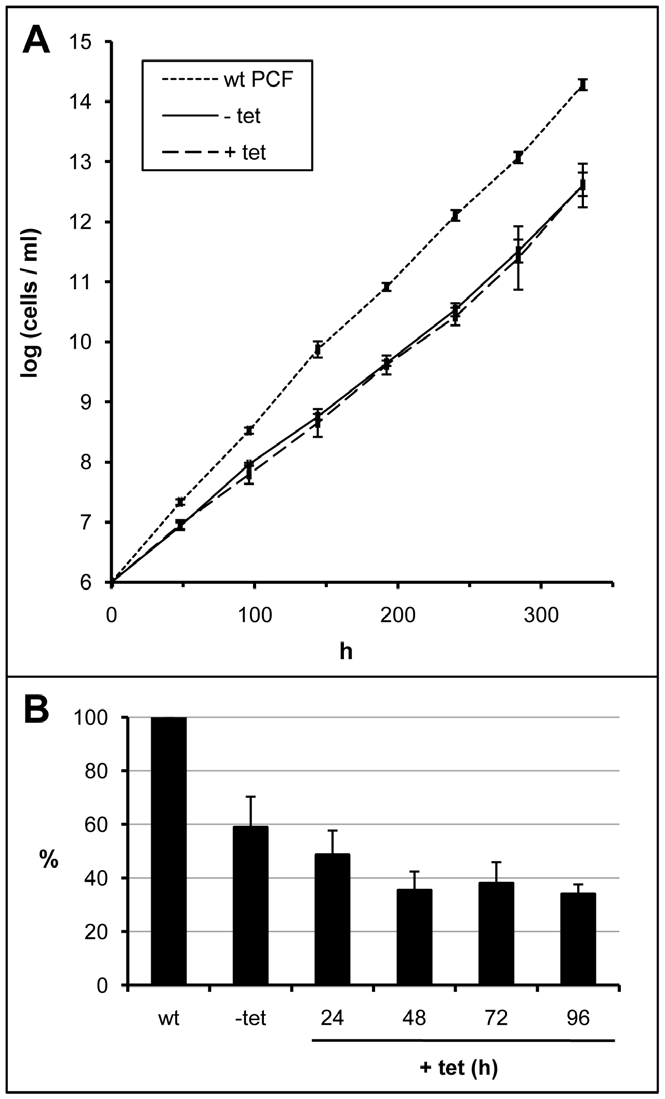
RNA interference of oleate desaturase expression in procyclic-form trypanosomes. Growth curves (logarithmic plot) of procyclic-form cell line 29-13 wild-type cells (wt PCF) and cells transfected with a construct for RNAi of OD. Cells of the transfected clone were cultured in the absence (-tet) and presence of tetracycline (+tet) to induce RNA interference (A). Relative levels of OD mRNA, normalized to tubulin mRNA levels. The results are expressed as percentage of the normalized OD transcripts present in the untransfected control (wt) cells and are the means ± SD of three independent experiments (B).

To confirm the essentiality of OD, its expression was knocked down by RNAi. To this end, plasmid constructs were prepared for expression of double-stranded RNA corresponding to the 5′-half of the *OD* structural gene. Linearized plasmid DNA, constitutively expressing the phleomycin-resistance marker gene, was stably integrated into the *T. brucei* genome. RNAi was induced by the addition of tet to the cultures, resulting in partial depletion of the targeted OD from the trypanosome cells. The growth rate of cells containing the RNAi construct was affected very early, even without induction with tet, indicating a leaky regulation of double-stranded RNA production from the T7 promoters in our system. Normal growth rate did not resume within 329 h but induced and non-induced cultures showed the same rate of growth, twofold lower than that of wild-type (wt, not transfected) cells (Fig. 3A). Levels of OD transcripts, normalized to the apparently stable mRNA levels of the non-targeted tubulin, were decreased even in non-induced cells, confirming the leaky regulation; an additional and progressive loss was evident from 24 to 96 h of growth after RNAi induction, below 40% of that in wild-type cells (Fig. 3B). The selection of phleomycin resistant cells was very difficult, yet cloned lines specifically and stably resistant to concentrations of this antibiotic that killed wild-type cells could be obtained (see Materials and Methods), but they all showed the leaky effect. These findings, together with the inability to completely knock down the expression of the gene and the drastic effect on growth rate, even considering that the enzyme activity was only partially ablated, indicate that OD is most probably essential in the PCF of *T. brucei*. The uptake of FAs from the culture medium was not effective in reverting such effect; supplementing the medium of the induced culture with linoleate was equally ineffective, like before for TS-treated cells (data not shown).

**Figure 4:**
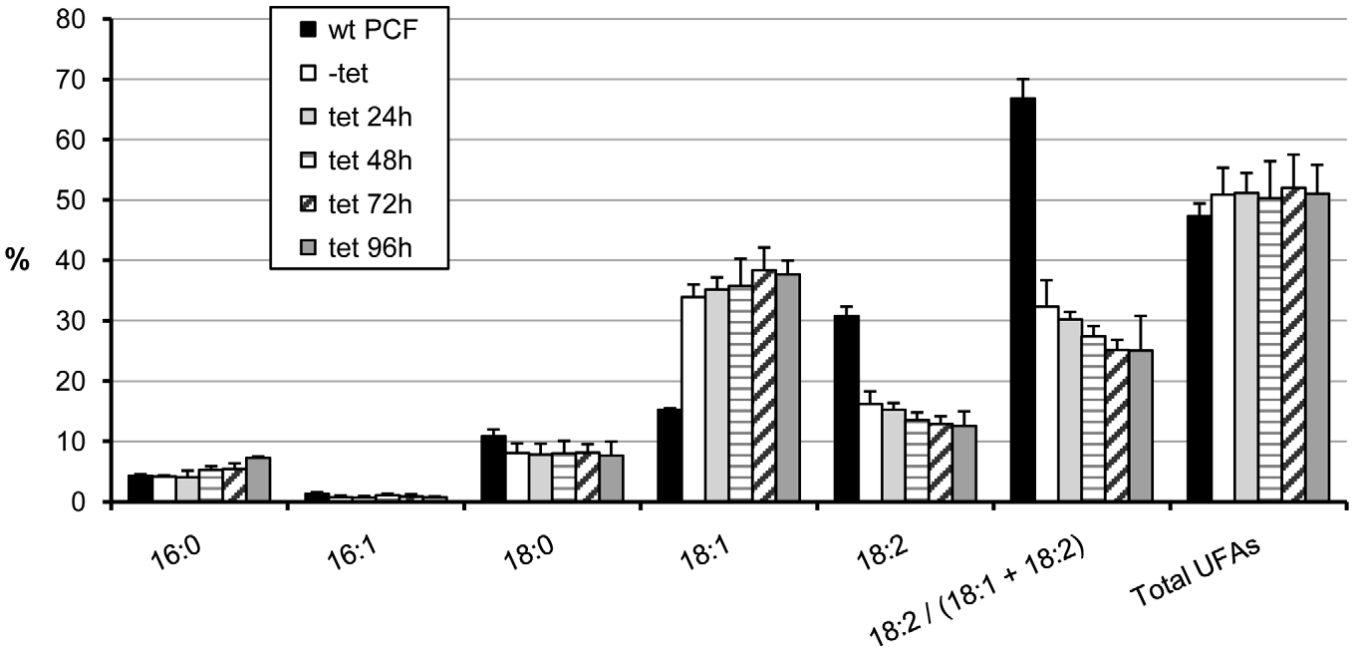
Changes of the fatty-acid profile of procyclic-form trypanosomes upon RNA interference of oleate desaturase expression. Procyclic cell line 29-13 (wt PCF) and the cell line containing the OD RNAi construct were grown in the absence (-tet) or presence (+tet) of tetracycline during the indicated time. The abundance of each fatty acid is presented as percentage of the total fatty acids. C16:0, palmitate: C16:1, palmitoleate; C18:0, stearate; C18:1, oleate; C18:2, linoleate; UFAs, unsaturated fatty acids. The results are means ± SD of three independent experiments.

The FAs profiles of cells were analyzed by GC-MS. As shown in Fig. 4, we found a drastic reduction of linoleate from 30.8% in wild-type to 16.2% in non-induced transfected cells. An additional and progressive drop was found till 12.6% after RNAi induction for 96 h. The conversion of oleate to linoleate was 66.8% in wild-type cells, 32.3% in non-induced transfected cells and 25% after 96 h induction. The stearate content was 10.8%, 8.1% and 7.6%, respectively for the same conditions. Interestingly, total unsaturated FAs were slightly increased, due to a considerable increase of oleate (from 15.3% to 34–38%), the substrate of OD, which was even larger than the decrease in linoleate level. This response of the cells to knocking down the OD expression is different from that reported above, observed after TS treatment of the trypanosomes, which reinforces the possibility that SCD was also partially inhibited by 12- and 13-TS. C16:0 showed a slight increase upon OD knockdown, which can be explained by assuming the absence of a requirement to increase this short FA for maintaining the physiological membrane fluidity, as the total unsaturated FAs remained near normal levels, even after 96 hours of RNAi.

### Effect of 12- and 13-thiastearates on bloodstream-form *T. brucei*

**Figure 5:**
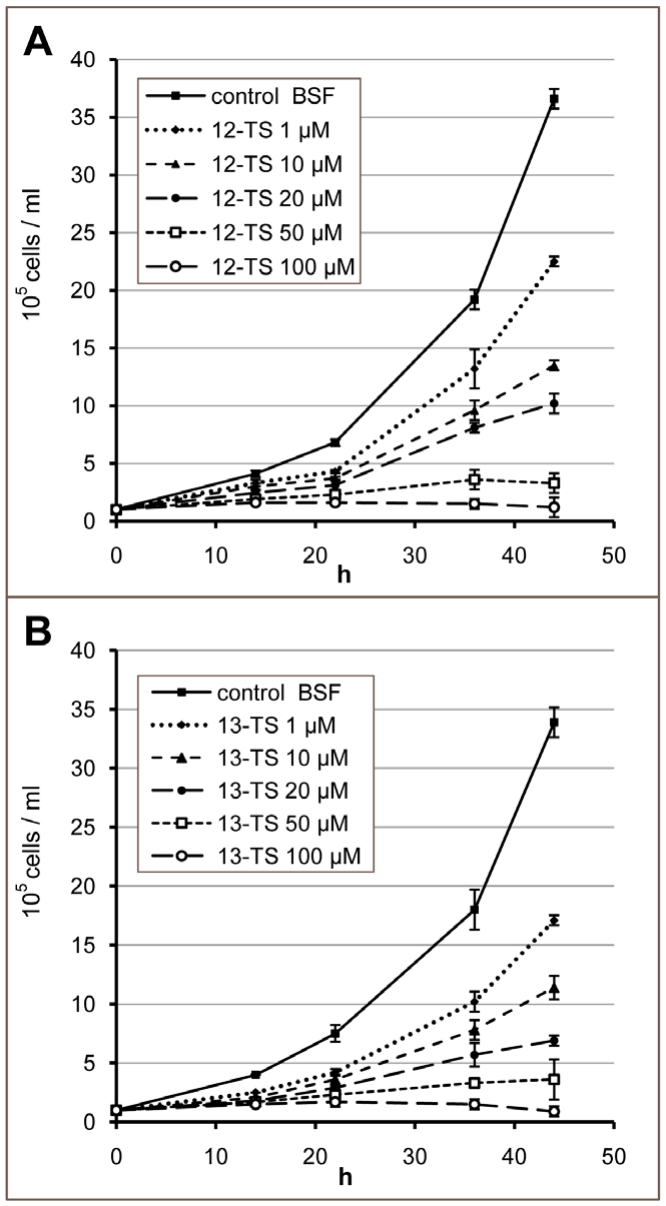
Effect of thiastearates on the growth of bloodstream-form *Trypanosoma brucei*. Growth curves are presented of BSF cells cultured in the absence (control BSF) or presence of increasing concentrations of 12-thiastearate (12-TS) (A) and 13-TS (B). The results are means ± SD of three independent experiments.

To evaluate *T. brucei* OD as a drug target, we tested the toxic effect of TSs on cultures of the mammalian infective BSF of the parasite. Fig. 5 shows that 50 µM of both TSs totally inhibited trypanosome growth, with EC_50_ values of 7±3 and 2±1 µM for 12- and 13-TS, respectively. Growth phenotype was not reverted by exogenous oleate or linoleate. Throughout the experiment the indicated concentration of drugs was maintained by adding them at each dilution of the culture, every 24 h. In a separate experiment, in which only one dose of drugs was added at the beginning of the growth of the cultures, inhibition was observed as well, although with an apparent EC_50_ of only 60±5 µM for 13-TS. The effect of 12- and 13-TS appears to be trypanocidal for both the BSF and PCF of *T. brucei*. By microscopal examination of the treated cultures, a decreased motility of the cells and abundant accumulation of cell debris were detected. Live cells with aberrant morphology were not particularly notorious as compared to non treated control cultures. It is interesting to note that treatment with the 10-TS isomer, which is a specific inhibitor of SCD (data not shown), produces a different phenotype, with an increased number of aberrant multiflagellated cells displaying reduced motility, and a very low cell-debris accumulation, most probably as consequence of a trypanostatic effect. It indicates that the phenotype is not related to the chemical nature of the used compound but to its target.

**Figure 6:**
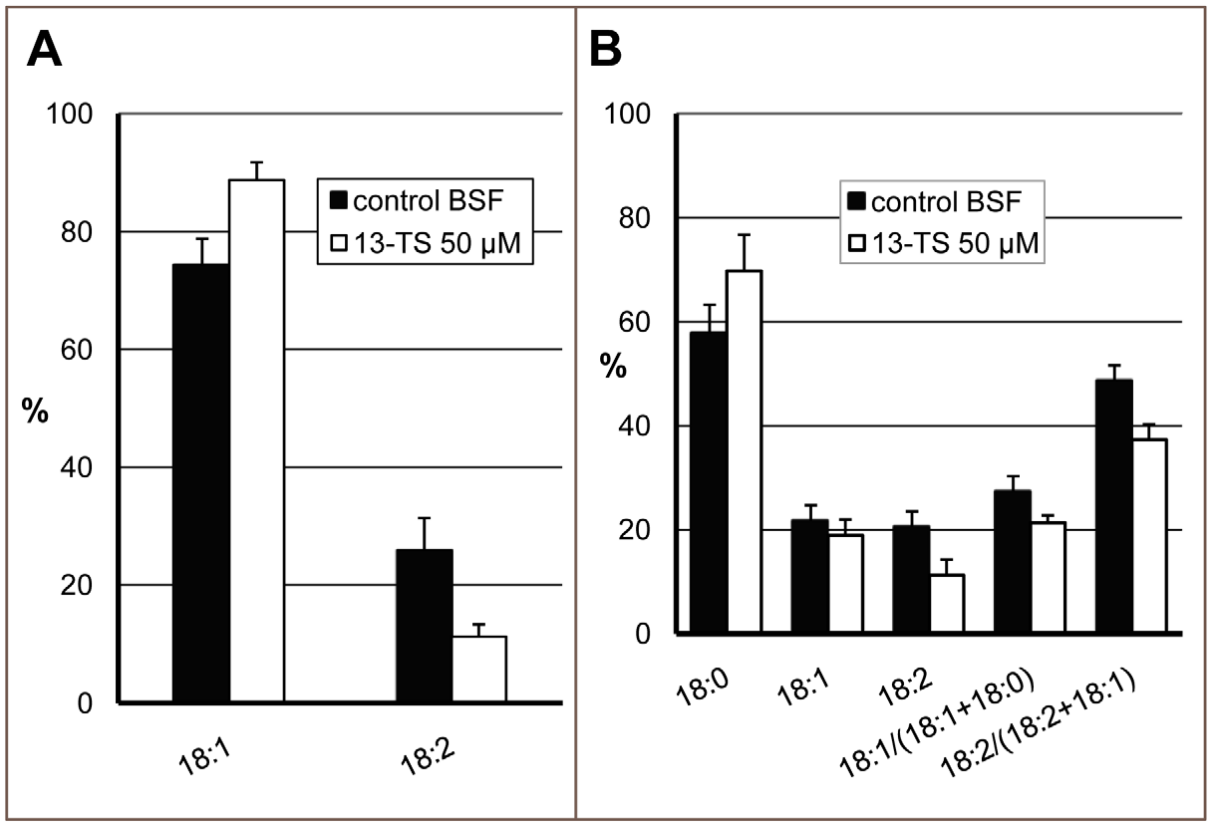
Effect of 13-thiastearate on the incorporation of radioactivity in membrane lipids of bloodstream-form *Trypanosoma brucei* from radiolabeled fatty acids. BSF cells were preincubated for 30 min with 50 µM 13-TS, then incubated with [^14^C]-oleate (A) or [^14^C]-stearate (B) for 1 h. The lipids were extracted by phase partitioning and fatty acids were analyzed by argentation-TLC after transmethylation, as described in Materials and Methods. The radioactive spots were scraped from the plate and quantified by liquid scintillation radioassay. The abundance of each fatty acid is presented as the percentage of sum of oleate (C18:1) and linoleate (C18:2) (A) or the sum of stearate (18:0), C18:1 and C18:2 (B). The results are means ± SD of three independent experiments.

As *T. brucei* BSF reaches the stationary phase of growth at a cell density one order of magnitude lower than PCF, analysis of the FA profile of the BSF cells by GC-MS was considered not realistic because of the high amounts of drugs that would be required. We have set up a whole cell radiolabeling assay to circumvent this experimental limitation. This method allows the analysis of the inhibitory effect of drugs within minutes instead of hours, identifying a direct effect on the putative targets before the cells react by modifying their FA composition to sustain normal membrane functions. *T. brucei* BSF cells (10^7^) were preincubated during 30 min with 50 µM of 13-TS, then incubated during 60 min with [^14^C]-oleate or [^14^C]-stearate. Total lipids were extracted and FAs analyzed by argentation-TLC after transmethylation. As shown in Fig. 6A, 13-TS strongly inhibited OD, as 20.4% of exogenous oleate was converted to linoleate in control cells whereas it dropped to 7.7% in treated cultures. A low but significant inhibition of SCD was detected as well, with a drop from 23.1% to 19% in the conversion of exogenous stearate to oleate (Fig. 6B).

### RNA interference of oleate desaturase in bloodstream-form *T. brucei*

**Figure 7:**
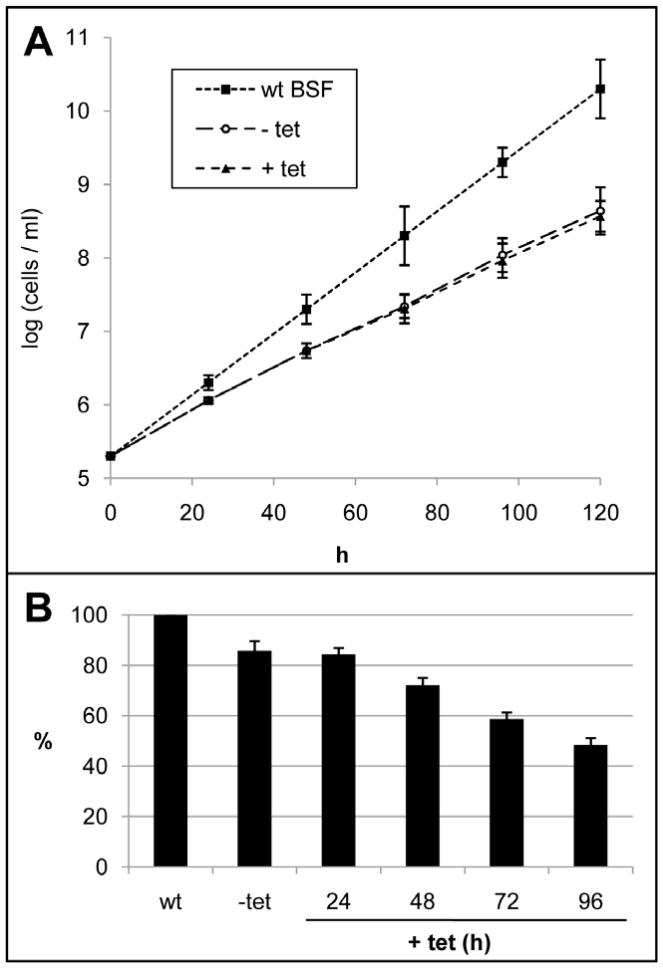
RNA interference of oleate desaturase expression in bloodstream-form trypanosomes. Growth curves (logarithmic plot) of bloodstream form cell line 90-13 wild-type (wt BSF) and cells transfected with a construct for RNAi of OD. Cells of the transfected clone were cultured in the absence (-tet) and presence of tetracycline (+tet) to induce RNAi (A). Relative levels of OD mRNA normalized to tubulin mRNA levels. The results are expressed as percentage of the normalized OD transcripts present in the untransfected control (wt) cells and are the means ± SD of three independent experiments (B).

**Figure 8:**
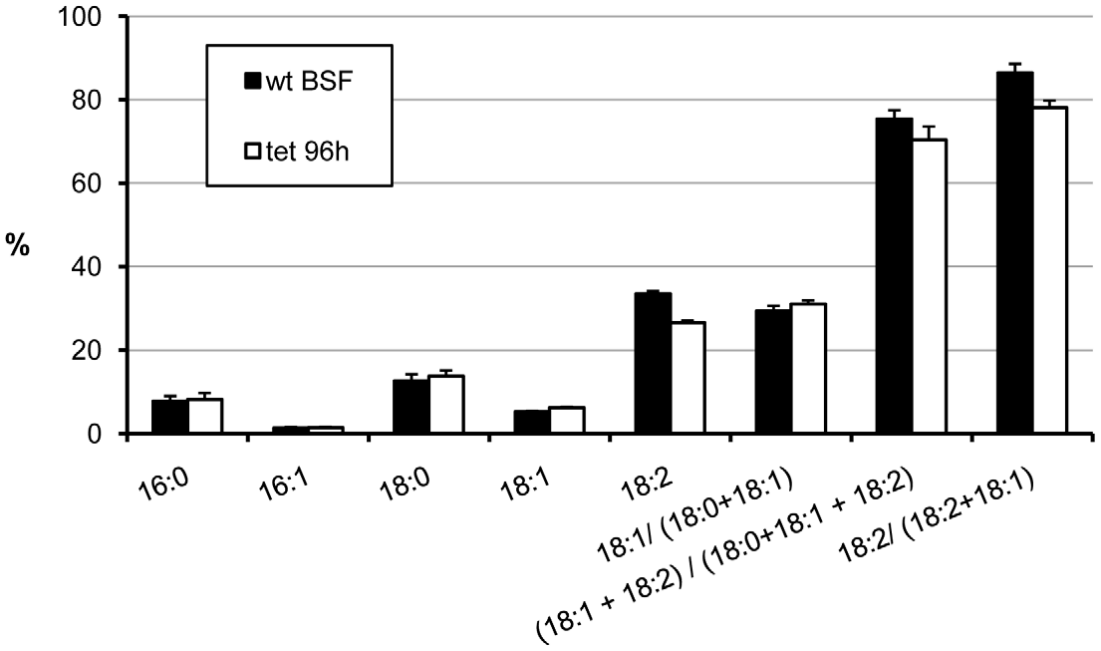
Changes of the fatty-acid profile of bloodstream-form trypanosomes upon RNA interference of oleate desaturase expression. Bloodstream-form cell line 90-13 (wt BSF) or the cell line containing the OD RNAi construct grown in the presence of tetracycline (tet) during 96 h. The abundance of each fatty acid is presented as percentage of the total fatty acids. C16:0, palmitate: C16:1, palmitoleate; C18:0, stearate; C18:1, oleate; C18:2, linoleate; UFAs, unsaturated fatty acids. The results are means ± SD of three independent experiments.

The experiments described above indicate that OD is expressed in BSF, and is essential for normal growth. In order to confirm it we knocked down the expression of OD in BSF cells in a similar way as indicated for PCF trypanosomes and by using the same DNA construction. Its insertion in the trypanosome's genome was evidenced by amplification of the phleomycin-*OD* gene cassette from purified genomic DNA, whereas no such amplification was found for control wild-type cells (not shown). The results are equivalent to those found for PCF cells. Again, selection of phleomycin resistant cells was difficult and cloned cell lines showed the leaky effect. Induced and noninduced cells presented identical growth rates, nearly twofold slower than wild-type cells (Fig. 7A). Although the level of OD mRNA, normalized to the tubulin mRNA level in the same samples, was always above 48% of that in wild-type cells (Fig. 7B) even after 96 h of induction, the content of linoleate was reduced from 33.5% to 26.5% (Fig. 8). It indicates that the ablation of OD is highly deleterious to the cell even when the activity was reduced by a mere 8%.

We assayed the effect of drugs on OD-knocked down cells after 72 h of RNAi induction by tetracycline. The EC_50_ values were 1.9±0.05 and 0.69±0.03 µM, respectively for 12-TS and 13-TS. It represents 3.7-2.9 fold lower values compared to those found for wild-type cells. In contrast, the known trypanocidal drug Suramin did not show exacerbated toxicity on interfered cells as compared to wild-type parasites (EC_50_ values of 0.38±0.06 and 0.40±0.07 µM, respectively). Such synergic effect between the thiastearates and RNAi is evidence that both treatments are having their impact on the same target.

## Discussion

Total FAs of *T. brucei* can be separated in two well differentiated pools by gas chromatography (Fig. 1): *pool* 1 which includes middle- and long-chain FAs, and *pool* 2 formed by C20 and C22 PUFAs with up to six double bonds. A fraction of the FAs present in *pool* 1 are those taken up from the culture media but a main part of them represent FAs synthesized *de novo* by the unprecedented pathway described in Introduction [3]. It implies the elongation of a C4 primer of unknown metabolic origin by the particulate ELO system, resulting in the synthesis of myristoyl-CoA, palmitoyl-CoA and stearoyl-CoA. The latter two acyl-CoAs are desaturated by SCD which is highly specific for stearoyl-CoA (Alloatti and Uttaro, unpublished results). Saturated and Δ9 desaturated moieties are then esterified to membrane lipids where oleoyl moieties are desaturated by OD, which is highly specific for oleate [11]. It results in the accumulation of linoleate, the most abundant FA found in trypanosomes. On the other hand, FAs present in *pool* 2 result only from the uptake and modification of FAs from the host or culture media and which are further desaturated and/or elongated by trypanosome's enzymes [8], [12]. The independence of both pools can be confirmed after inhibition of OD, as the sum of FAs in *pool* 2 remains invariable, around 25% (Table 1) or after knocking down the enzyme (not shown).

Membrane fluidity is mainly regulated by balancing the proportion of unsaturated and saturated FAs of phospholipids. This parameter is essential to maintain the correct functionality of the bilayer, highlighting SCD and OD as key enzymes for such regulation. Our RNAi experiments indicate that OD is expressed in both life-cycle stages of the parasite (PCF and BSF) and that a partial inhibition of its expression produced drastic effects on growth (Fig. 3 and 7). Similar effects were seen by knocking down the SCD (Gupta et al, unpublished results), indicating that both desaturases are promising targets for chemical intervention, in particular OD, as mammals lack this activity. Our RNAi experiments on PCF are particularly informative with regard to the basic lipid composition for normal membrane functionality. One would expect that a deleterious effect on growth would result from a change in membrane fluidity due to a drop of total unsaturated FAs. Growth of PCF cells in which OD synthesis was targeted by RNAi was indeed affected, but the total pool of unsaturated FAs was maintained at a constant level, but with a decreased linoleate and increased oleate content. This indicates that not only fluidity but a normal content in one of the membrane components (linoleate) could be essential for such functionality. We have previously found that 12- and 13-TS specifically inhibit *T. cruzi* OD and growth of epimastigote forms of the parasite [13]. Our results indicate that the *T. brucei* BSF was more sensitive to these drugs, as 50 µM was sufficient to completely inhibit its growth, with EC_50_ values of 7±3 and 2±1 µM, respectively (Fig. 5), nearly one order of magnitude lower than that found for the PCF (this work) or *T. cruzi* epimastigotes [13]. However, *T. brucei* SCD was slightly inhibited by the TS as well, causing oleate to remain constant at the expense of a considerable drop of linoleate, as shown for PCF cells in Table I. This could explain part of the parasite's growth sensitivity to TSs. The decreased linoleate content in TS-treated cells may have caused the cells to attempt maintaining the membrane fluidity by increasing their level of shorter saturated FAs, notably 16:0.

The combination of such sensitive target and the use of drugs like thiastearates, which are of relatively simple chemical synthesis and low toxicity [14], [26], could be particularly useful, because it may allow developing countries to produce locally the drugs in an economically interesting manner, for treatment of these neglected diseases.

An additional consequence of our RNAi experiments was the confirmation of the identity of the OD gene, which we have previously identified after transfection of yeast with it and biochemical characterization of the expressed protein [11]. One important conclusion of our previous work was that cytochrome *b_5_* is the electron donor for the desaturase reaction and that the cytochrome *b*-like domain of SCD can act as an alternative electron donor. As our results show that OD is active in the BSF, it is indicative that a cytochrome *b_5_* or the equivalent domain of *T. brucei* SCD (which is equally active in the BSF) has to be functional in transferring electrons. For a number of years, it was believed that BSF trypanosomes lack cytochromes. However, Vanhollebeke et al. [27] demonstrated recently in *T. brucei* BSF the presence of receptors specifically designed to acquire heme by internalizing hemoproteins from the host plasma.
